# Determining the indirect costs of suicide in Sweden: a national population-based cross-sectional study, 2010–2019

**DOI:** 10.1136/bmjopen-2024-095174

**Published:** 2025-09-28

**Authors:** Daniela Wikström, Camilla Nystrand, Gergö Hadlaczky, Filip Gedin

**Affiliations:** 1 Department of Learning, Informatics, Management and Ethics, Karolinska Institutet, Stockholm, Sweden; 2 Center for Health economics, informatics and health care research, Stockholm Healthcare Services, Stockholm, Stockholm, Stockholm, Sweden; 3 Department of Clinical Neuroscience, Karolinska Institutet, Stockholm, Sweden

**Keywords:** Public health, HEALTH ECONOMICS, Suicide & self-harm, PREVENTIVE MEDICINE

## Abstract

**Abstract:**

**Objectives:**

Globally, more than 700 000 people commit suicide annually. In Sweden, the yearly incidence ranges between 1000 and 1500 people, which is higher than the global average. The aim of this study is to estimate the economic burden related to indirect costs that suicide has imposed on Swedish society between 2010 and 2019.

**Design:**

National population-based cross-sectional study.

**Participants and setting:**

All suicides in Sweden between 2010 and 2019, using data from the Swedish National Cause of Death Registry.

**Outcomes:**

Indirect costs associated with suicides, estimated using the human capital approach, including productivity loss over 1-year and lifetime horizons.

**Results:**

Between 2010 and 2019, 1406 to 1591 suicides occurred annually in Sweden, resulting in approximately 26 500 productive life years lost each year. In 2019, the productivity loss due to suicides was estimated at €44 million over a 1-year horizon and €935 million over a lifetime horizon. The corresponding per-person costs were €37 000 and €778 000, respectively.

**Conclusions:**

This study provides valuable insights into the economic burden of suicide on Swedish society. It underlines the potential economic benefits of effective suicide prevention, aligning with previous research highlighting the substantial returns—both monetary and in terms of human well-being—that successful prevention strategies can yield.

**STRENGTHS AND LIMITATIONS OF THIS STUDY**

- Uses high-quality nationwide registries, eliminating selection bias.
- Comprehensive analysis due to the inclusion of all suicide cases in Sweden.
- Conservative assumptions are likely to lead to an underestimation of the total cost burden.
- Excludes individuals aged 67+ years, potentially missing some individuals who are still active in the workforce.
- Relies on aggregate data, which may reduce the precision of estimates.

## Introduction

 More than 700 000 people commit suicide across the world annually,^1^ and between 1000 and 1500 people commit suicide per year in Sweden.^2^ The suicide rate is greater in Sweden than in the rest of the globe, with 11.7 per 100 000 deaths compared with 8.8 deaths globally.^3^ People aged above 65 years are more likely to commit suicide, followed by people aged between 45 and 64 years.^2^ Common risk factors for suicides in Sweden include psychiatric disorders,^4^ substance use disorders,^5^ social isolation^6^ and various stressful life events.^7^ Among adults, men are 2.5 times more likely to commit suicide than women are, and in the 15- to 29-year-old group, suicide is one of the leading causes of death.^1^ Suicide is classified as a major public health problem, not only because a person dies prematurely but also because of its extensive implications for family members, relatives, friends and society at large. Although it is well known that mental health issues such as psychotic disorders and mood and anxiety disorders are associated with an increased risk for suicide, a recent meta-analysis estimated that the population attributable risk of mental disorders as a whole was approximately 21%.^8^ This substantial proportion should not be downplayed, but it is perhaps not surprising that most working-age individuals who die by suicide have no underlying mental health disorder and have an occupation at the time of their death.^9^

Indirect costs, such as productivity losses, far exceed direct medical costs.^10 11^ By highlighting these costs, we can underscore the significant economic impact of suicide on society. This economic perspective can be a compelling argument for investing in preventive measures, as it demonstrates the broader societal benefits of reducing suicide rates. However, these cost estimations are not based on complete national registries, introducing uncertainty in the estimations. While Sweden has some of the world’s most comprehensive individual-level registries, it allows for encompassing estimations of the burden of suicide.

Understanding the economic burden of productivity losses caused by suicide could help highlight the importance and urgency of finding more effective treatments and preventative measures to help people suffering from suicidal thoughts and support studies evaluating the cost-effectiveness of various interventions. In addition, how the economic burden is spread across different actors in society is important to policymakers and for budget allocation because it provides arguments for the potential economic benefit for different actors to invest in preventive measures. The aim of this study is to estimate the economic burden related to indirect costs that suicide has imposed on Swedish society between 2010 and 2019.

## Methods

This national population-based cross-sectional study estimated the indirect costs associated with all suicides in Sweden between 2010 and 2019. Indirect costs were estimated using the human capital approach, which considers productivity losses due to premature death. This method was chosen because it provides a comprehensive estimate of the economic impact of suicide by capturing the total earnings lost, including the portions that would have been paid as taxes and social contributions. This approach highlights the substantial economic burden of suicide on society, reinforcing the need for effective preventive measures. This study was conducted in accordance with the Consolidated Health Economic Evaluation Reporting Standards reporting guidelines (see online supplemental table 1). All data used in the study have been aggregated from publicly available data. Hence, no ethical approval or consent has been necessary. Excel was used for all statistical analyses.

### Data collection

Data regarding the number of suicides committed in Sweden per year were acquired from the Swedish National Cause of Death Registry, which is held by the Swedish National Board of Health and Welfare. The data were accessed for research purposes from 2010 to 2019. The authors did not have access to information that could identify individual participants during or after data collection. To approximate the true incidence of suicides in Sweden, suicide deaths classified as International Classification of Diseases (ICDs) X60–X84/E950–E959 were combined with so-called uncertain suicides, that is, ICD Y10–Y34/E980–E989, between 2010 and 2019. The data were divided into yearly cohorts based on the year when the suicide occurred. Each yearly cohort was further subgrouped based on sex and age.

The parameters included in the calculations for productivity loss are outlined in table 1. These included the average salary in Sweden retrieved from Statistics Sweden and data on average employment, social fees, the pension rate and vacation compensation. Furthermore, table 1 displays the division of income taxes paid to the regional and local authorities.

**Table 1:** Parameters included in the calculations

Parameter	Estimation	Sources
Average monthly salary in Sweden 2019	€3100	17
Average employment rate in Sweden 2019	67.3%	18
Social fees	31.42%	19
Pension fee	4.5%	13
Vacation compensation	12%	20
Taxes on salary compensation		
Local level authority tax	20.70%	21
Regional level authority tax	11.49%	21
Funeral fee	0.23%	21
Conversion from SEK to Euros	1 SEK=€0087*	22

* Exchange rate on 26 November 2023.

SEK, Swedish Krona.

### Cost analysis

#### Productivity loss

The human capital approach was used to estimate the productivity loss that occurs due to premature death following completed suicide. Years of productive life lost were estimated by subtracting the age when the suicide occurred from the retirement age in Sweden and including the average mortality rate for each age (probability of surviving another year).

Productivity loss was calculated over two different time horizons: the year following the death and over the lifetime. Estimations were performed in total numbers for the yearly cohorts as well as per person. Productivity loss was further estimated over five different age groups, 18–24, 25–34, 35–44, 45–54 and 55–67, both over a 1-year time horizon and over a lifetime. The parameters included in the estimations were the average employment rate in Sweden, the average monthly salary, social fees (31.42% of the monthly salary), vacation compensation (12% of the monthly salary) and the pension fee paid by employers (4.5% of the monthly salary); see table 1. All calculations were performed on a yearly basis.

Due to premature death and loss of productivity, individuals are unable to contribute to the tax system at large. Taxes in Sweden are divided into state-, regional- and local-level authorities. Most of these taxes are paid by individuals through salary compensation and value-added tax on goods and services purchased and by employers through social and pension fees. Tax loss (part of the total indirect cost) was estimated as the percentage of the monthly income that would have gone to taxes, with a regional tax of 11.49% of the monthly income and a local authority tax of 20.70% of the monthly salary.

### Discounting, economic growth and inflation

For the lifetime horizon, future costs were discounted at an annual rate of 3%, which is recommended for health economic evaluations in Sweden.^12^ This discount rate accounts for the time preference of money, reflecting the principle that costs incurred in the future are less valuable than costs incurred today. Additionally, future economic growth rates and inflation were considered by adjusting the average salary growth rate based on historical data from Statistics Sweden. The average annual growth rate of salaries was assumed to be 2%, and the inflation rate was assumed to be 1.5%, based on historical trends.

### Net versus gross salary

The salary data used in this study represent gross salaries, which include all taxes and social fees. The salaries were adjusted for age-specific variations. This approach provides a comprehensive estimate of the economic impact of suicide by capturing the total earnings lost, including the portions that would have been paid as taxes and social contributions.

### Sensitivity analysis

In the sensitivity analysis, the employment rate was varied to 40%, 50%, 60%, 70% and 80% to account for the possible difference in productivity for this specific cohort. Another sensitivity analysis was conducted for the 2019 cohort, where it was assumed that the person died 6 months into the year, instead of at the beginning of the year.

### Patient and public involvement

No patients or members of the public were involved in the study.

## Results

Between 2010 and 2019, 1406 to 1591 suicides occurred every year in Sweden. The average age of people who committed suicide during these years was approximately 50 years (table 2). In total, approximately 26 500 productive life years were lost every year due to suicide (online supplemental table 2).

**Table 2:** Demographic characteristics of suicides in Sweden (2010–2019).

	Total number of suicides (certain)	Sex		Age				
		Male	Female	0–24	25–44	45–64	65+	Average age
2010	1446 (1138)	1033	413	151	364	579	352	51
2011	1406 (1110)	982	424	161	413	516	316	49
2012	1538 (1158)	1080	458	154	394	613	377	51
2013	1620 (1236)	1117	503	183	438	583	416	50
2014	1548 (1161)	1058	490	177	445	540	386	50
2015	1571 (1192)	1105	466	155	452	576	387	50
2016	1494 (1146)	1024	470	137	440	522	395	51
2017	1554 (1200)	1070	484	158	459	518	417	51
2018	1591 (1280)	1098	493	172	492	526	394	50
2019	1588 (1269)	1078	510	168	498	525	397	49

### Total cost

Between 2010 and 2019, the total cost of suicide related to indirect costs varied between €32 million and €44 million over a 1-year horizon and between €795 million and €935 million over a lifetime horizon. The indirect costs per person varied between €28 000 and €37 000 over a 1-year horizon and between €708 000 and €778 000 over a lifetime horizon (table 3).

**Table 3:** Total forgone costs due to suicide over a 1-year time horizon and a lifetime horizon

Year	Productivity losses (in Euros)		Productivity losses per person (in Euros)	
	1 year	Lifetime	1 year	Lifetime
2010	32 000 000	795 000 000	28 000	708 000
2011	33 000 000	830 000 000	30 000	758 000
2012	37 000 000	865 000 000	31 000	725 000
2013	38 000 000	926 000 000	31 000	760 000
2014	38 000 000	909 000 000	32 000	769 000
2015	38 000 000	891 000 000	32 000	743 000
2016	38 000 000	834 000 000	34 000	751 000
2017	41 000 000	883 000 000	35 000	769 000
2018	44 000 000	926 000 000	37 000	769 000
2019	44 000 000	935 000 000	37 000	778 000

Exchange rate of SEK to Euros on 26 November 2023. 1 SEK=€0.087.

SEK, Swedish Krona.

### Age groups

The productivity loss between different age groups was estimated (online supplemental table 3), both in total and per person. Over a 1-year period, the highest total and per-person costs were found for the 45- to 54-year-old age group, followed by the 35- to 44-year-old age group. The cost per person for the 45- to 54-year-old age group ranged between €54 000 and €56 000. The lowest costs are found in the 18–24 age group; in this age group, the costs vary between €17 000 and €19 000 per person.

Over a lifetime horizon (table 4), the highest cost is found for the 18–24 age group per person. For this group, the costs vary by €1.6 million. The lowest costs are found in the 55- to 67-year-old age group, where the costs vary by €280 000.

**Table 4:** Total cost per person for different age groups over a lifetime horizon

Age group	Productivity loss over a lifetime (in Euros)	Productivity loss per person over a lifetime (in Euros)
18–24	227 000 000	1 600 000
25–34	411 000 000	1 500 000
35–44	262 000 000	1 200 000
45–54	192 000 000	720 000
55–67	72 000 000	280 000

### Loss of taxes 2019

As part of the indirect costs of suicide in 2019, the losses in taxes for different sectors amounted to approximately €6.4 million for the local authorities and €3.6 million for the regional authorities. The lost social fees that would have been transferred to the state for the cohort that passed away in 2019 amount to €14.5 million.

### Sensitivity analysis

In the sensitivity analysis, the employment rate varied between 40% and 80%. The average employment rate in Sweden is approximately 67%. In 2019, the indirect costs over a 1-year period varied between €20 000 and €40 000 per person. Over a lifetime, the costs varied between €428 000 and €856 000. In the different age groups, the indirect cost (per person) over a 1-year period varied between €15 000 and €31 000 (age group 18–24), €18 000–€37 000 (age group 25–34), €22 000–€44 000 (age group 35–44), €23 000–€46 000 (age group 45–54) and €22 000–€45 000 (age group 55–67). Over a lifetime horizon, the indirect costs due to suicide (per person) varied between €734 000 and €1.5 million (age group 18–24), €655 000–€1.3 million (age group 25–34), €507 000–€1 million (age group 35–44), €323 000–€647 000 (age group 45–54) and €131 000–€262 000 (age group 55–67). An additional sensitivity analysis was performed where it was assumed that the person died 6 months into the year, instead of at the start of the year. In this sensitivity analysis, the production loss was calculated over 6 months in 2019, and the indirect cost amounted to €18 500 during the year 2019. Over a lifetime perspective, the indirect cost for this person would amount to approximately €760 000.

## Discussion

This study provides valuable insights into the economic burden of productivity losses caused by suicide in Swedish society between 2010 and 2019. During this period, the annual number of suicides ranged from 1406 to 1591, with an average age of approximately 50 years. The findings reveal substantial financial implications, with suicide accounting for significant amounts in terms of both 1-year and lifetime estimates. In 2019, the total productivity losses amounted to €44 million annually. These losses were distributed across various sectors. The state incurred €14.5 million, regional authorities €3.6 million and local authorities €6.4 million in tax losses.

The indirect cost varied significantly across different demographic groups. The highest total and per-person costs were found for the 46–54 age group, followed by the 35–44 age group. For the 45–54 age group, the cost per person ranged between €54 000 and €56 000 over a 1-year period. In contrast, the lowest costs were found in the 18–24 age group, with costs varying between €17 000 and €19 000 per person. Over a lifetime horizon, the highest costs were observed in the 18–24 age group, with costs per person reaching approximately €1.6 million.

The costs per person, though varying across different studies, offer a comparative perspective. A study from Finland estimated substantially lower costs per person over a lifetime perspective than reported in this study (€322 928 compared with €729 575). The reason for this is the different methods used in the estimation. We estimated indirect costs based on loss of production, whereas Solin and colleagues measured costs only in terms of lost tax returns in Finland.^11^ In contrast, an Australian study which estimated indirect costs of suicide for individuals aged 24 years and younger found a similar yearly indirect cost, €1 700 000, compared with our estimates of €1 600 000 for individuals aged 24 years and younger.^13^

This study underlines the potential economic benefits of effective suicide prevention, aligning with previous research highlighting the substantial returns, both monetary and in terms of human well-being, that successful prevention strategies can yield. The economic foundation provided by this study can provide powerful support for knowledge-based decision-making as well as for the development and evaluation of preventive measures.

The focus on costs associated with loss of productivity excludes other substantial costs related to suicide, such as emergency response, medical care, family members’ incapacity for work and/or potential treatment costs related to complicated grief and other disorders of relatives, costs related to the transport system (eg, in railway, subway and motorway suicides) and property damage. Both Kinchin *et al* and Solin *et al* included costs related to direct medical or emergency expenditures.^11 14^ However, the direct cost only accounted for a fraction of the total cost of suicide, and the other unrelated costs are also likely to be only a fraction of the total costs related to suicide. In the Kinchin *et al* study, healthcare costs during suicide-related emergencies only accounted for 0.01% of the total cost, and in the study by Solin *et al*, the productivity loss from deceased people accounted for approximately 70%. Similarly, a study by Segar *et al*, estimating the costs of suicides in France, found that the direct costs of completed suicides are very small compared with indirect costs, with direct costs accounting for only 0.4%, highlighting the significant economic burden of productivity losses and other societal impacts.^15^

In this study, we have chosen to focus exclusively on completed suicides for several reasons. First, completed suicides are clearly defined and recorded in national databases, providing us with accurate and reliable data, which minimises the risk of inaccuracies and uncertainties in our analyses. Second, completed suicides have a direct and measurable economic impact on society, including lost productivity and tax revenues. By focusing on these, we can clearly demonstrate the economic burden and the need for effective preventive measures. Third, policymakers and decision-makers require concrete and specific data to make informed decisions about resource allocation and the development of preventive strategies. By focusing on completed suicides, we provide information needed to prioritise preventive interventions that may save lives. By limiting our scope to completed suicides, we can conduct a more focused and in-depth analysis. Lastly, previous studies in this field have also focused on completed suicides.^11 14^ By doing the same, the results are comparable to previous research and contribute to a more coherent and comparable knowledge base.

This study has several strengths. First, it uses data from high-quality nationwide registries, which eliminates selection bias and ensures that all cases of suicide in Sweden are included in the dataset. Second, the complete dataset allows for a comprehensive analysis of the data, which is a major strength of the study. Third, the assumptions made in the study are conservative, which means that the results are probably an underestimation of the total cost burden.

The study also has some limitations. One limitation is the decision to only include individuals aged 18–67 years, based on presumed employment, which raises concerns about the exclusion of a significant portion of the population. The age for retirement in Sweden is 67 years, and it is common to assume that income and tax-generating productivity are subsequently reduced to zero. However, this group, particularly the 85+ years group, has the highest suicide risk among all age groups, and most individuals who retire in Sweden continue to contribute to unpaid work. However, the consequence of using the human capital approach for estimating the costs of suicide is the exclusion of this group from the analysis. Another limitation is the precision of our estimates. The Swedish nationwide registers used as data inputs were on an aggregate level; hence, the employment and salary for each individual were not available, and average estimates had to be used. These limitations should be kept in mind when interpreting the findings of this study. Finally, we included both certain and uncertain suicides in the estimation of productivity losses. Some of the uncertain suicides may not have been suicides, which may have led to an overestimation of the total indirect costs of suicide.

Using secondary data in this study presents some limitations. First, it lacks detail and precision, as it does not account for individual differences in income, employment status or occupation type, which are important for estimating productivity loss. Second, the economic impact estimations might be imprecise because aggregated data often only provide average age bands or income brackets, leading to rough and potentially biased estimates. Lastly, there’s the issue of not being able to adjust for other factors that might influence productivity loss, which means the analysis might incorrectly attribute all differences to suicide. These limitations suggest that the findings should be interpreted with caution and highlight the need for more detailed data in future research.

The findings of this study underscore the substantial economic burden that suicide imposes on Swedish society, with total productivity losses amounting to €44 million annually. This significant financial impact highlights the urgent need for more suicide prevention initiatives and research. Policymakers should prioritise the allocation of resources towards mental health services and suicide prevention programmes, particularly targeting high-risk demographic groups. By investing in preventive measures, not only can we reduce the human toll of suicide, but we can also achieve considerable economic savings. The economic argument presented in this study provides a compelling case for the development and implementation of comprehensive suicide prevention policies, which can lead to substantial returns in terms of both monetary savings and improvements in public health and well-being.

### Policy implication

The results of this study are important at all levels of society. At the national level, our results can inform the allocation of resources towards nationwide suicide prevention strategies and mental health initiatives. At the regional and local levels, the data can help tailor interventions to specific communities, ensuring that resources are directed where they are most needed. Local authorities can use this information to develop targeted programmes that address the unique needs of their populations, potentially leading to more effective and efficient use of funds.

In Sweden, suicide prevention efforts are coordinated at the national level by the Public Health Agency, which collaborates with various stakeholders to develop and disseminate knowledge and preventive measures. While these efforts primarily focus on public health strategies, there are also initiatives aimed at reducing indirect costs through workplace programmes. For example, employers can play a vital role in suicide prevention by integrating mental health support into workplace culture, offering programmes that prioritise employee well-being and fostering a supportive environment. These workplace initiatives can significantly reduce productivity losses and other indirect costs associated with suicide.^16^ By promoting mental health and providing support for employees at risk, employers can contribute to the overall reduction of suicide rates and the associated economic burden.

Patient support groups can use our findings to advocate for better mental health services and suicide prevention programmes. By presenting the economic impact of suicide, these groups can make a compelling case to policymakers and stakeholders about the importance of investing in mental health resources. Additionally, support groups can use this data to raise awareness about the broader societal costs of suicide, encouraging community involvement and support for preventive measures.

## Conclusion

This study sheds light on the economic burden of suicide in Sweden, emphasising the need for comprehensive preventive strategies. The study provides a nuanced understanding of the economic implications of completed suicides, laying the groundwork for future research and policy discussions in the realm of suicide prevention.

## Supplementary material

10.1136/bmjopen-2024-095174 online supplemental file 1

## Data Availability

Data are available upon reasonable request and with the permission of National Centre for Suicide Research and Prevention (NASP).

## References

[1] Organization TWH (2024). Suicide.

[2] lll-Health NCfSRaPoM Interpreting suicide data 2023.

[3] Dattani S, Rodés-Guirao L, Ritchie H (2023). Suicides.

[4] Reutfors J, Brandt L, Ekbom A (2010). Suicide and hospitalization for mental disorders in Sweden: a population-based case-control study. J Psychiatr Res.

[5] Crump C, Sundquist K, Sundquist J (2014). Sociodemographic, psychiatric and somatic risk factors for suicide: a Swedish national cohort study. Psychol Med.

[6] Hedstrom P, Liu K-Y, Nordvik MK (2008). Interaction Domains and Suicide: A Population-based Panel Study of Suicides in Stockholm, 1991-1999. *Social Forces*.

[7] Mallon S, Galway K, Hughes L (2016). An exploration of integrated data on the social dynamics of suicide among women. Sociol Health Illn.

[8] Too LS, Spittal MJ, Bugeja L (2019). The association between mental disorders and suicide: A systematic review and meta-analysis of record linkage studies. J Affect Disord.

[9] Matilla Santander N, Blazevska B, Carli V (2022). Relation between occupation, gender dominance in the occupation and workplace and suicide in Sweden: a longitudinal study. BMJ Open.

[10] Shepard DS, Gurewich D, Lwin AK (2016). Suicide and Suicidal Attempts in the United States: Costs and Policy Implications. Suicide Life Threat Behav.

[11] Solin P, Tamminen N, Seppänen A (2022). Calculation of Costs Related to Death by Suicide in Finland. Soc Sci (Basel).

[12] (TLV) T-ol (2017). Allmänna råd om ekonomiska utvärderingar (TLVAR 2017:1).

[13] Swedish Tax Agency (2023). Anställd inom kommun och region. https://www.pensionsmyndigheten.se/forsta-din-pension/tjanstepension/anstalld-inom-kommun-och-region.

[14] Kinchin I, Doran CM (2018). The Cost of Youth Suicide in Australia. Int J Environ Res Public Health.

[15] Segar LB, Laidi C, Godin O (2024). The cost of illness and burden of suicide and suicide attempts in France. BMC Psychiatry.

[16] Hallett N, Rees H, Hannah F (2024). Workplace interventions to prevent suicide: A scoping review. PLoS One.

[17] Swedish National Mediation Office (2020). Löneskillnaden Mellan Kvinnor Och Män 2019: Vad Säger Den Officiella Lönestatistiken.

[18] SCB (2020). Arbetskraftsundersökningarna (AKU) 2019 [Elektronisk Resurs].

[19] Skatteverket (2019). Arbetsgivaravgifter.

[20] Mo L (1985). The Swedish annual leave act.

[21] Statistics Sweden The total municipal tax rate increases next year: Statistics Sweden.

[22] European central bank (ECB) (2024). Euro foreign exchange reference rates: ECB.

