# Supplementary material for: Determining the indirect costs of suicide in Sweden: a national population-based cross-sectional study, 2010–2019
Source: BMJ Open. 2025 Sep 28;15(9):e095174. doi: 10.1136/bmjopen-2024-095174 (PMC12481339; doi:10.1136/bmjopen-2024-095174)
Supplement: Supplementary file 2 [file bmjopen-2024-095174.reviewer_comments.pdf]

PEER REVIEW HISTORY

BMJ Open publishes all reviews undertaken for accepted manuscripts. Reviewers are asked to complete a checklist review form and are provided with free text boxes to elaborate on their assessment. These free text comments are reproduced below.

ARTICLE DETAILS

Title (Provisional)

Determining the indirect costs of suicide in Sweden, 2010-2019 - A cost of illness study

Authors

Wikström, Daniela; Nystrand, Camilla; Hadlaczky, Gergö; Gedin, Filip

---

VERSION 1 - REVIEW

**Reviewer**	**1**
**Name**	**Prasartpornsirichoke, Jirada**
**Affiliation**	**Chulalongkorn University, Psychiatry**
**Date**	**22-Dec-2024**
**COI**	**None**

Determining the indirect cost of suicide: A cost of illness study – A cost of illness study

This study aims to quantify the indirect costs of suicide to Swedish society from 2010 to 2019. This study utilized secondary data on the number of deaths attributed to suicide causes (certain suicide (intentional self-harm); ICDs X60-X84/E950-E959 and uncertain suicide (events of undetermined intent); Y10-Y34/E980-989) from the Swedish National Cause of Death Registry to estimate the indirect costs of suicide employing the human capital approach. Furthermore, the researchers indicated that indirect costs pertain to the loss of productivity resulting from premature death due to suicide. The productivity loss was assessed over two distinct timeframes: the year subsequent to death and throughout the individual's lifetime, accounting for the remaining years of potential productivity adjusted by the annual mortality rate. The index employed by the researchers to calculate productivity loss comprised the average monthly salary in Sweden for 2019, the average employment rate in Sweden for 2019, social fees, pension contributions, vacation compensation, and taxes on salary compensation (including local authority tax, regional tax, and funeral fees). The annual discount rate was presumed to be 3%. The annual salary growth rate was given at 2%, while the inflation rate was projected at 1.5%. The researchers employed sensitivity analysis to evaluate various scenarios of the employment situation in Sweden.

This study's strength resides in its provision of the indirect costs of suicide in Swedish society from 2010 to 2019, utilizing high-quality nationwide macro-level data. Upon completing the reading, I have several comments, as follows:

Introduction part

1. The phrase 'incidence of suicide' may not be appropriate in this context. Refer to the suicide rate, as the original data source is more precise. The incidence rate is calculated by dividing the number of new cases by the population at risk.
2. Kindly verify the statistical data regarding the suicide rates in Sweden (10.7 deaths) and globally (9 deaths). The suicide rate's year and the date of data retrieval are not specified. I doublechecked the data from the reference section's cited source, but the statistics are inaccurate.
3. Indeed, nearly all developed nations exhibit higher suicide rates than the global average. Kindly furnish more detailed information to comprehend the suicide circumstances within Swedish society.
4. The economic burden pertains to both direct and indirect costs associated with suicide. This study solely addressed productivity loss resulting from suicide. It is advisable to employ consistent terminology in the study.

Method part

5. The analysis exhibited an ambiguous understanding of the indirect costs associated with suicide. The researchers did not clarify the perspective considered for estimating the indirect costs, whether from the standpoint of society, a public health provider, a patient and family, or an employer, in the methodology section. The study's perspective influences the estimation and composition of the indirect costs associated with suicide.

6. The researchers indicated that they employed the human capital approach to estimate productivity loss resulting from suicide. This method employed the current market price of gross salary to assess productivity loss on the day of illness. This study utilized the average monthly salary in Sweden for 2019 and a discount rate for calculations rather than employing actual data from 2010 to 2018. Moreover, utilizing the average monthly salary without accounting for years of experience, particularly among individuals over 50 who anticipate having a higher average salary, correlates significantly with the prevalence of suicide. This causes underestimation of the analysis.
7. Kindly provide additional details regarding the salary system in Sweden. In my view, the pre-tax and pre-pension deduction earnings of Swedish workers determine the average monthly salary. From the reader's perspective, this suggests a double calculation. This leads to an overestimation of the analysis. Furthermore, I believe that the employer should not include the pension fee in the calculation of productivity loss.
8. Providing baseline characteristics of suicide for each year, including sex, age range, and cause of death (ICDs), will enhance readers' comprehension of the manuscript's content.
9. The researchers should not depend on an assumed index set from 2019. A distinction should exist between suicides occurring in early January and those occurring in late December.

Discussion part

1. Kindly provide more discussion on the significant findings based on your own dataset.

**Reviewer**	**2**
**Name**	**Durand-Zaleski, Isabelle**
**Affiliation**	**University of Paris**
**Date**	**18-Feb-2025**
**COI**	**None**

The objective of the study is to estimate to indirect costs of suicide in Sweden. The methods are well described and adequate, the results are clearly presented.

I would like to suggest that the authors need to better explain how their results can be used to shape policy.

1) The reason to focus on indirect costs could be explained in more detail

2) how can their results be used by policy makers to design and implement prevention policies

3) how are the results useful at the national, regional, local levels

4) are some preventive measures in Sweden or other countries specifically targeted at the reduction of indirect costs (programs developed by employers?)

5) how can patients support group use the results for advocacy

VERSION 1 - AUTHOR RESPONSE

Reviewer: 1
Introduction

1. *The phrase 'incidence of suicide' may not be appropriate in this context. Refer to the suicide rate, as the original data source is more precise. The incidence rate is calculated by dividing the number of new cases by the population at risk.*

Response: we agree that suicide rates are better than suicide incidence and will revise the text accordingly.

Revised text: (Introduction, Paragraph 1) The **suicide rate** is greater in Sweden than in the rest of the globe, with 10.7 deaths compared to 9 deaths per 100,000 people on average.

2. *Kindly verify the statistical data regarding the suicide rates in Sweden (10.7 deaths) and globally (9 deaths). The suicide rate's year and the date of data retrieval are not specified. I double-checked the data from the reference section's cited source, but the statistics are inaccurate.*

Response: you are correct and we are sorry to report the wrong numbers. We have adjusted this in the text accordingly.

Revised text: (Introduction, Paragraph 1) The suicide rate is greater in Sweden than in the rest of the globe, with **11.7 per 100,000 deaths** compared to **8.8 deaths** globally[3].

3. *Indeed, nearly all developed nations exhibit higher suicide rates than the global average. Kindly furnish more detailed information to comprehend the suicide circumstances within Swedish society.*

Response: We really appreciate this question! As the reviewer probably suspects it is difficult to explain with certainty the reason behind developed nations' higher suicide rates than the global average. The causes of suicide are highly complex and difficult to interpret as testified by a metaanalysis of 50 years of research by Franklin et al (2016). Franklin et al found around 4 000 effectsizes on a broad range of risk-factors, but concluded that despite knowledge about these risk-factors, even complex models of suicidal behaviours have an extremely low explanatory value. It is probably safe to say that a common denominator in most suicides are suffering and hopelessness (no matter where on the globe), but the pathways that lead to these are complex and heterogenous- and its difficult to say in what way Swedish suicides are unique. With that said we've added a sentence that describes common risk factors in Sweden:

Revised text: (Introduction, Paragraph 1) **Common risk factors for suicides in Sweden include psychiatric disorders[4], substance use disorders [5], social isolation [6], and various stressful life events[7].**

4. *The economic burden pertains to both direct and indirect costs associated with suicide. This study solely addressed productivity loss resulting from suicide. It is advisable to employ consistent terminology in the study.*

Response: Thank you for this comment, we agree that the total economic burden of suicide is not covered by only measuring the productivity losses. We have made changes to the text, both in the introduction but also in the discussion, to make this clear.

Revision to the text:

(Introduction, Paragraph 3) Understanding the economic burden **of productivity losses caused by** suicide could help highlight the importance and urgency of finding more effective treatments and preventative measures to help people suffering from suicidal thoughts and support studies evaluating the cost-effectiveness of various interventions.

(Discussion, Paragraph 1) This study provides valuable insights into the economic burden **of productivity losses by** caused by suicide on Swedish society.

Method part

5. *The analysis exhibited an ambiguous understanding of the indirect costs associated with suicide. The researchers did not clarify the perspective considered for estimating the indirect costs, whether from the standpoint of society, a public health provider, a patient and family, or an employer, in the methodology section. The study's perspective influences the estimation and composition of the indirect costs associated with suicide.*

Response: thank you for highlighting this error. This should be clearly stated in the method. We have revised the method accordingly.

Revision to the text: This national population-based cross-sectional study estimated the indirect costs associated with all suicides in Sweden between 2010 and 2019. Indirect costs were estimated using the human capital approach **from a societal perspective**.

6. *The researchers indicated that they employed the human capital approach to estimate productivity loss resulting from suicide. This method employed the current market price of gross salary to assess productivity loss on the day of illness. This study utilized the average monthly salary in Sweden for 2019 and a discount rate for calculations rather than employing actual data from 2010 to 2018. Moreover, utilizing the average monthly salary without accounting for years of experience, particularly among individuals over 50 who anticipate having a higher average salary, correlates significantly with the prevalence of suicide. This causes underestimation of the analysis.*

Response: We appreciate your concerns regarding the use of average monthly salary without accounting for years of experience, particularly among individuals over 50. However, if wages are used to calculate the value of production, they should be standard or mean wages. Group differences in wage rates can arise on other grounds than the actual value of production (Swedish Council on Health Technology Assessment, n.d.). For example, there are differences in wage rates between men and women. If production by men and women is valued differently based on official data, a treatment aimed at men could result in a lower ICER than the same treatment for women, leading to higher priority for treatments aimed at men. To avoid this, production can be valued at the average national wage rate instead of a gender or age-specific average.

Swedish Council on Health Technology Assessment (SBU). (n.d.). Metodboken: En introduktion till evidensbaserad medicin. Available at: https://www.sbu.se/contentassets/76adf07e270c48efaf67e3b560b7c59c/eng_metodboken.pdf [Accessed 4 Mar. 2025].

7. *Kindly provide additional details regarding the salary system in Sweden. In my view, the pre-tax and pre-pension deduction earnings of Swedish workers determine the average monthly salary. From the reader's perspective, this suggests a double calculation. This leads to an overestimation of the analysis. Furthermore, I believe that the employer should not include the pension fee in the calculation of productivity loss.*

Response: We appreciate your feedback. In Sweden, the average monthly salary is reported as gross earnings before tax and pension deductions. Our calculation of productivity loss was based on these gross salary figures to provide a comprehensive measure of economic impact. Excluding pension fees from the calculation would underestimate the true cost of productivity loss, as pensions are a significant component of total compensation. Our approach aligns with standard economic evaluation practices (Swedish Council on Health Technology Assessment), ensuring that our results are comparable to other studies in the field. Including the full gross salary, including pension contributions, provides a more accurate representation of the economic impact of productivity loss due to suicide.

Swedish Council on Health Technology Assessment (SBU). Assessment of methods in health care and social services: A handbook. Available at: https://www.sbu.se/contentassets/76adf07e270c48efaf67e3b560b7c59c/eng_metodboken.pdf

8. *Providing baseline characteristics of suicide for each year, including sex, age range, and cause of death (ICDs), will enhance readers' comprehension of the manuscript's content.*

Response: We agree that including a table with baseline characteristics would enhance the readers comprehension of the manuscript's content. Therefore, we have added a table to the result.

Revision to the text: Please see table 2.

9. *The researchers should not depend on an assumed index set from 2019. A distinction should exist between suicides occurring in early January and those occurring in late December.*

Response: Thank you for your comment. We understand your concern regarding the use of an assumed index set from 2019. However, it is important to note that suicide rates typically peak twice a year, in spring and autumn, with the rest of the cases spread throughout the year. This pattern has been observed in both Finland and Sweden (Holopainen et al., 2013). Given this seasonal variation, distinguishing between suicides occurring in early January and those in late December may not significantly impact the overall analysis.

To address your concern, we will include a sensitivity analysis. This will help ensure that our estimates are robust and account for potential variations.

Revision to the text: (Result, sensitivity analysis, Paragraph 1) **An additional sensitivity analysis was performed where it was assumed that the person died six months into the year, instead of at the start of the year. In this sensitivity analysis the production loss was calculated over six months in 2019, and the indirect cost amounted to 18 500 euros during the year 2019. Over a lifetime perspective the indirect cost for this person would amount to approximately 760 thousand euros.**

Holopainen, J., Helama, S., Björkenstam, C. and Partonen, T., 2013. Variation and seasonal patterns of suicide mortality in Finland and Sweden since the 1750s. Environmental Health and Preventive Medicine, 18(6), pp.494-501. doi: 10.1007/s12199-013-0348-4.

Discussion part

10. *Kindly provide more discussion on the significant findings based on your own dataset.*

Response: Thank you for your valuable feedback. We appreciate your suggestion to provide more discussion on the significant findings based on our dataset. We have revised the discussion section to address this comment and have highlighted the changes in bold.

Revision to the text:

(Discussion, Paragraph 1). Our study provides valuable insights into the economic burden of productivity losses caused by suicide in Sweden between 2010 and 2019. **During this period, the annual number of suicides ranged from 1,406 to 1,591, with an average age of approximately 50 years.** The total productivity losses due to suicides amounted to **44 million euros annually** in 2019, highlighting the significant financial impact on Swedish society. **These losses were distributed across various sectors, with the state incurring 14.5 million euros, regional authorities 3.6 million euros, and local authorities 6.4 million euros in tax losses.**

(Discussion, Paragraph 2) **The economic burden varied significantly across different demographic groups. The highest total and per-person costs were found for the 45-54 age group, followed by the 35-44 age group. For the 45-54 age group, the cost per person ranged between 54,000 euros and 56,000 euros over a one-year period. In contrast, the lowest costs were found in the 18-24 age group, with costs varying between 17,000 euros and 19,000 euros per person. Over a lifetime horizon, the highest costs were observed in the 18-24 age group, with costs per person reaching approximately 1.6 million euros.**

(Discussion, Paragraph 8) **The findings of this study underscore the substantial economic burden that suicide imposes on Swedish society, with total productivity losses amounting to 44 million euros annually. This significant financial impact highlights the urgent need for more suicide prevention initiatives and research. Policymakers should prioritize the allocation of resources towards mental health services and suicide prevention programs, particularly targeting highrisk demographic groups. By investing in preventive measures, not only can we reduce the human toll of suicide, but we can also achieve considerable economic savings. The economic argument presented in this study provides a compelling case for the development and implementation of comprehensive suicide prevention policies, which can lead to substantial returns in terms of both monetary savings and improvements in public health and well-being.**

Reviewer: 2

1. *The reason to focus on indirect costs could be explained in more detail*

Response: We appreciate the reviewer's comment and have revised the introduction to provide a more detailed explanation of the focus on indirect costs.

Revision to the text:

(Introduction, 2 paragraph) **Indirect costs, such as productivity losses, far exceed direct medical costs [6 7]. By highlighting these costs, we can underscore the significant economic impact of suicide on society. This economic perspective can be a compelling argument for investing in preventive measures, as it demonstrates the broader societal benefits of reducing suicide rates.** However, these cost estimations are not based on complete national registries, introducing uncertainty in the estimations. While Sweden has some of the world's most comprehensive individual-level registries, it allows for encompassing estimations of the burden of suicide.

(Method, 1 paragraph) This national population-based cross-sectional study estimated the indirect costs associated with all suicides in Sweden between 2010 and 2019. **Indirect costs were estimated using the human capital approach, which considers productivity losses due to premature death. This method was chosen because it provides a comprehensive estimate of the economic impact of suicide by capturing the total earnings lost, including the portions that would have been paid as taxes and social contributions. This approach highlights the**

substantial economic burden of suicide on society, reinforcing the need for effective preventive measures. This study was conducted in accordance with the CHEERS reporting guidelines (see Supplementary Table 1). All data used in the study have been aggregated, publicly available data. Hence no ethical approval or consent has been necessary.

2. *how can their results be used by policy makers to design and implement prevention policies*

Response: We appreciate the reviewer's comment and have revised the manuscript to explain how the results can be used by policymakers. The results of our study on the indirect costs of suicide can be highly valuable for policymakers in designing and implementing prevention policies. By highlighting the substantial economic burden of suicide, primarily driven by productivity losses, our findings provide a compelling argument for investing in preventive measures. Policymakers can use this information to prioritize resource allocation, ensuring that funds are directed towards effective mental health services and public awareness campaigns. Integrating these insights into broader public health strategies can enhance clinical management and community-based interventions, ultimately reducing the human and economic toll of suicide.

Revision to the text: (Discussion, Paragraph 8) The findings of this study underscore the substantial economic burden that suicide imposes on Swedish society, with total productivity losses amounting to 44 million euros annually. This significant financial impact highlights the urgent need for more suicide prevention initiatives and research. **Policymakers should prioritize the allocation of resources towards mental health services and suicide prevention programs, particularly targeting high-risk demographic groups. By investing in preventive measures, not only can we reduce the human toll of suicide, but we can also achieve considerable economic savings. The economic argument presented in this study provides a compelling case for the development and implementation of comprehensive suicide prevention policies, which can lead to substantial returns in terms of both monetary savings and improvements in public health and well-being.**

3. *how are the results useful at the national, regional, local levels*

Response: We appreciate the reviewer's comment. At the national level, our results can inform the allocation of resources towards nationwide suicide prevention strategies and mental health initiatives. At the regional and local levels, the data can help tailor interventions to specific communities, ensuring that resources are directed where they are most needed. Local authorities can use this information to develop targeted programs that address the unique needs of their populations, potentially leading to more effective and efficient use of funds.

4. *are some preventive measures in Sweden or other countries specifically targeted at the reduction of indirect costs (programs developed by employers?)*

Response: We appreciate the reviewer's comment. In Sweden, suicide prevention efforts are coordinated at the national level by the Public Health Agency, which collaborates with various stakeholders to develop and disseminate knowledge and preventive measures. While these efforts primarily focus on public health strategies, there are also initiatives aimed at reducing indirect costs through workplace programs. For example, employers can play a vital role in suicide prevention by integrating mental health support into workplace culture, offering programs that prioritize employee well-being, and fostering a supportive environment. These workplace initiatives can significantly reduce productivity losses and other indirect costs associated with

suicide. By promoting mental health and providing support for employees at risk, employers can contribute to the overall reduction of suicide rates and the associated economic burden.

5. *how can patients support group use the results for advocacy*

Response: Patient support groups can use our findings to advocate for better mental health services and suicide prevention programs. By presenting the economic impact of suicide, these groups can make a compelling case to policymakers and stakeholders about the importance of investing in mental health resources. Additionally, support groups can use this data to raise awareness about the broader societal costs of suicide, encouraging community involvement and support for preventive measures.

VERSION 2 - REVIEW

**Reviewer**	**1**
**Name**	**Prasartpornsirichoke, Jirada**
**Affiliation**	**Chulalongkorn University, Psychiatry**
**Date**	**10-Apr-2025**
**COI**	

---

1. Kindly specify the statistical software utilized for the calculation of indirect costs and the conduct of the sensitivity analysis.

2. Lines 216–218: The details regarding the additional sensitivity analysis should be included in the Methods section (line 166).

3. Lines 192–194: Please recheck the accuracy of the currency units reported in this section.

4. Please discuss the limitations associated with the use of secondary data in this study.

VERSION 2 - AUTHOR RESPONSE

Reviewer: 1

1. *Kindly specify the statistical software utilized for the calculation of indirect costs and the conduct of the sensitivity analysis.*

Response: Thank you for pointing this out and we have revised the text accordingly.

Revised text: (Method, first paragraph last sentence) Excel was used for all statistical analyses.

2. *Lines 216–218: The details regarding the additional sensitivity analysis should be included in the Methods section (line 166).*

Response: thank you for this comment, we agree that this should be clearly described in the methods and have added a description to the method on the added sensitivity analysis.

Revised text: (Method, sensitivity analysis) Another sensitivity analysis was conducted, for the 2019 cohort, where it was assumed that the person died six months into the year, instead of at the beginning of the year.

3. Lines 192–194: Please recheck the accuracy of the currency units reported in this section.

Response: We greatly appreciate that this error was found. We have now corrected this mistake in the text.

4. Please discuss the limitations associated with the use of secondary data in this study.

Response: we agree that there is a need to address the limitations of secondary data in this study presents. We have included this to our discussion on limitations.

Revised text: (Discussion, 9th paragraph)

Using secondary data in this study present some limitations. Firstly, it lacks detail and precision, as it doesn't account for individual differences in income, employment status, or occupation type, which are important for estimating productivity loss. Secondly, the economic impact estimations might be imprecise because aggregated data often only provide average age bands or income brackets, leading to rough and potentially biased estimates. Lastly, there's the issue of not being able to adjust for other factors that might influence productivity loss, which means the analysis might incorrectly attribute all differences to suicide. These limitations suggest that the findings should be interpreted with caution and highlight the need for more detailed data in future research.
